# Comparison of SuperDARN peak electron density estimates based on elevation angle measurements to ionosonde and incoherent scatter radar measurements

**DOI:** 10.1186/s40623-020-01170-w

**Published:** 2020-03-31

**Authors:** Alexander V. Koustov, Sydney Ullrich, Pavlo V. Ponomarenko, Robert G. Gillies, David R. Themens, Nozomu Nishitani

**Affiliations:** 1 Department of Physics and Engineering Physics, University of Saskatchewan, Saskatoon, SK Canada; 2 Department of Physics and Astronomy, University of Calgary, Calgary, AB Canada; 3 Department of Physics, University of New Brunswick, Fredericton, NB Canada; 4 Institute for Space-Earth Environmental Research, Nagoya University, Nagoya, Aichi Japan

**Keywords:** F region electron density, SuperDARN radars, Elevation angles, CADI ionosonde, RISR incoherent scatter radar

## Abstract

Measurements of the electron density at the F region peak by the Canadian Advanced Digital Ionosonde (CADI) and the Resolute Incoherent Scatter Radar (RISR) are used to assess the quality of peak electron density estimates made from elevation angle measurements by the Super Dual Auroral Radar Network (SuperDARN) high-frequency radar at Rankin Inlet (RKN). All three instruments monitor the ionosphere near Resolute Bay. The CADI-RKN joint dataset comprises measurements between 2008 and 2017 while RISR-RKN dataset covers about 60 daylong events in 2016. Reasonable agreement between the RKN estimates and measurements by CADI and RISR is shown. Two minor discrepancies are discussed: RKN radar daytime peak electron density overestimation by ~ 10% and underestimation by up to 30% in other time sectors. In winter nighttime and dawn, cases were identified in which the RKN radar significantly overestimates the peak electron density. This occurs when the phase in the RKN interferometer measurements is incorrectly shifted by $2\uppi$, and this is most significant when electron densities are low. Statistical fitting to the joint data sets, split into four time sectors of a day, has been done and parameters of the fit have been determined. These allow slight adjustment of measured real-time RKN values to better reflect real peak electron densities in the ionosphere within its field of view.
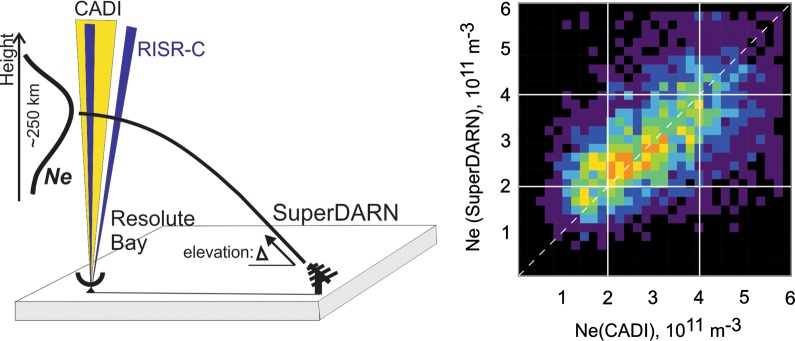

## Introduction

Knowledge of the electron density distribution in the high-latitude ionosphere is fundamentally important for various practical applications such as high-frequency (HF) radio wave communication (Davies 1990; Hunsucker 1991; Rawer 2013). At HF, radio waves experience significant ionospheric refraction resulting in strong bending of radio paths and occasionally turning radio waves toward the ground where they can be either detected by a communication receiver or can be backscattered by the surface and detected by a ground-based radar receiver near their location of transmission.

Decades of research has led to a general understanding of the horizontal and vertical distributions of the electron density in the ionosphere, culminating in the development of empirical models of the ionosphere such as the International Reference Ionosphere (IRI, Bilitza et al. 2017) and the Empirical Canadian High Arctic Ionospheric Model (E-CHAIM, Themens et al. 2017, 2019), which is an improved empirical model for high-latitude regions.

It is well established that the ionospheric F region has the largest electron density and thus affects HF radio waves in the most significant way. The electron density at the F layer maximum $N_{\text{m}} F2$ has always been of special interest, both experimentally and in the theoretical modeling of the physical processes leading to ionosphere formation (e.g., Kutiev et al. 2013). Despite significant progress in this area, numerous details and trends in the electron density distribution require further investigation so that forecasting capabilities can be improved.

The electron density at the F region peak has been traditionally studied through ionosonde observations (Davies 1990; Hunsucker 1991; Rawer 2013). This is because obtaining the maximum electron density value from routinely recorded ionograms is a relatively easy task provided that the ionogram traces are well defined. Ionosonde data are usually available with 1 to 15 min resolution. Over the years, a significant body of data, covering a wide range of latitudes, has been accumulated (e.g., https://www.sws.bom.gov.au/World_Data_Centre).

The electron density distribution with height, along with the F layer peak values, is also routinely retrieved from incoherent scatter radar (ISR) measurements (Beynon and Williams 1978; Hunsucker 1991; Rawer 2013). Modern phased-array ISRs sound the ionosphere along several beams nearly simultaneously, allowing them to build a 3-D distribution of the electron density with temporal resolutions often as good as 1 min (e.g., Gillies et al. 2016). One important aspect of both ionosonde and ISR operations is that the region of measurements is reasonably constrained and known. This is necessary for multi-instrument studies, and for the development of ionospheric models, such as the IRI series (Bilitza 2018), E-CHAIM and others (e.g., Themens et al. 2017, 2019). However, for some applications a global coverage is highly desired. Despite the continuously growing array of ionosondes and ISRs, their numbers are still limited and achieving global coverage for instantaneous electron density measurements is still a challenging task.

Relatively recently, the Super Dual Auroral Radar Network (SuperDARN) radars, which operate within the HF band, have been used for measurements of the F region peak electron density (André et al. 1998; Bland et al. 2014; Ponomarenko et al. 2011). To make electron density estimates, elevation angles of echo arrival are considered. Both types of SuperDARN echoes, ionospheric scatter (IS) and ground scatter (GS), have been used for electron density measurements. Identification of the ionospheric region for the electron density estimates is less certain than with the ionosondes and ISRs. For the work with GS signals, the region of strong radio wave bending (leading to radio waves turning toward the ground) can be as large as several hundred kilometers. For IS signals, the estimates rely on the occurrence of Pedersen rays (Davies 1990) that reach the top of the ionospheric layer and return with about the same elevation angle from a number of ranges (Ponomarenko et al. 2011). The algorithm by Ponomarenko et al. (2011) requires an echoing region with Pedersen rays extending at least ~ 200 km.

Although the possibility of producing peak electron density measurements with the SuperDARN radars has been known for years, the method has not been regularly implemented and very limited testing of the method has been done so far (André et al. 1998; Ponomarenko et al. 2011). Part of the problem is the need for reliable echo elevation angle measurements which require difficult calibration of phased arrays in the HF band (e.g., Ponomarenko et al. 2015, 2018). Testing has also been limited by the need for an independent instrument measuring the electron density distribution in the ionosphere within the radar field of view. Very few SuperDARN radars have ionosondes or ISRs positioned at ranges where electron densities are typically obtained.

The SuperDARN radar at Rankin Inlet (RKN) is one of a few radars that has reliable elevation angle data for many years of operation (Chisham 2018; Ponomarenko et al. 2011). Continuous operation of the Canadian Advanced Digital Ionosonde (CADI) at Resolute Bay (Jayachandran et al. 2009) and the recent deployment of an ISR at the same location (to be referred to as RISR) provide an excellent opportunity for testing the quality of electron density estimates from RKN observations near the Resolute Bay zenith.

This study expands the initial work by Ponomarenko et al. (2011) by performing a multi-year and two-instrument comparison of the F region peak electron density estimates from RKN observations of ionospheric echoes.

## SuperDARN method of F region peak electron density estimates from elevation angle measurements

SuperDARN HF radar waves transmitted into the ionosphere experience strong refraction that depends on the vertical distribution of the electron density. An important result of the refraction is that radio waves can propagate almost perpendicular to the geomagnetic field lines in extended regions of the high-latitude F layer, often stretching from 700 to 1200 km in range. In this “quasi-orthogonal” radio wave propagation, the presence of field-aligned decameter irregularities allows for return signals detectable by radar. Of special interest are ionospheric signals corresponding to Pedersen rays. For Pedersen rays, typically coming from above the F layer maximum, the returned elevation angles are about the same irrespective of the radar range. One important aspect here is that elevation angles for Pedersen rays and those coming from the maximum of the F layer differ insignificantly, on the order of ~ 1°–2°. This can be shown by ray tracing for typical ionospheric conditions, see for example Greenwald et al. (2017), Figs. 3 and 4.

**Fig. 1:**
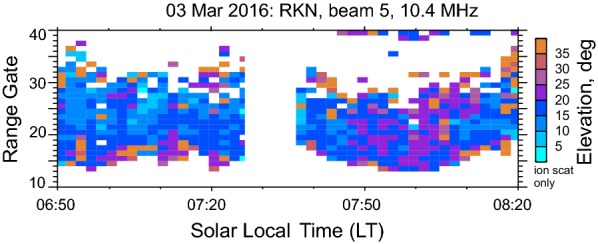
An example of elevation angle data for the RKN radar recorded on 03 March 2016. Corrections of the instrumental time delay of 3 ns have been applied. The nearly constant elevation angles, used for electron density estimates, are colored in blue and violet. Notice presence of gates with high-elevation angles (brown color) mixed with low elevation angles (blue color) at large range gates

Figure 1 gives an example of SuperDARN elevation angle data taken from RKN radar observations on 03 March 2016. To plot these data, an instrumental delay of 3 ns was applied. For all other data considered in this study, the delays were computed as described in Ponomarenko et al. (2015). In Fig. 1, the elevation angles do not change much with range gate in many instances, as evidenced by the dominating blue and violet color. These measurements correspond to Pedersen ray detection. There are also high-elevation echoes, colored in brown, at far ranges of the echo band. These are mixed with low elevation (blue) echoes. The echoes with the lowest elevation angles at the far edge of echo bands are typical and are expected if the height of the scatter does not change much. The occurrence of high-elevation echoes is sporadic. This is a nonphysical effect caused by the radar software flipping the phase difference between the main and interferometer arrays by $2\uppi$ (e.g., Milan et al. 1997; Ponomarenko et al. 2018).

Ponomarenko et al. (2011) suggested estimating the electron density at the F region maximum $N_{\text{m}} F2$ by measuring the elevation angle of Pedersen rays. In the Ponomarenko et al. ’s (2011) technique (we will call it “the P2011 technique”) it is assumed that the ionosphere is spherically stratified and symmetric so that Snell’s law for the rays launched at an elevation angle $\Delta$ (at the radar site) and reaching the orthogonality condition in the ionosphere at the height of $h_{\text{s}}$ can be written in the form (Gillies et al. 2009):

1 $$ \text{cos}\Delta = \frac{{R_{\text{E}} + h_{\text{s}} }}{{R_{\text{E}} }}n_{\text{S}} \, \text{sin}\gamma , $$

where $n_{\text{S}}$ is the refractive index of the ionospheric plasma, $R_{\text{E}}$ is the Earth’s radius (with Earth assumed to be a sphere of the radius of 6370 km) and $\gamma$ is the angle of the magnetic field line (within the scattering volume) with respect to the horizontal plane. Since the index of refraction for HF radio waves at frequency $f_{0}$ is related to the plasma frequency $f_{\text{p}}$ as

2 $$ f_{\text{p}} = f_{0} \sqrt {1 - n_{\text{S}}^{2} } , $$

the electron density at the F2 layer maximum can be evaluated from

3 $$ N_{\text{m}} F2 = \frac{{4\uppi^{2} m\varepsilon_{0} f_{\text{p}}^{2} }}{{e^{2} }}, $$

where *m* and *e* are the electron mass and charge and $\varepsilon_{0}$ is the permittivity of free space.

Determination of $N_{\text{m}} F2$ is done by finding the nearly constant elevation angles $\Delta$ that indicate the occurrence of Pedersen rays. In the algorithm implemented in the present study, the P2011 technique was used. Five sequential range gates with about the same elevation angles anywhere between range gates 15 and 40 were used to identify occurrences of Pedersen rays. Similar to P2011, we determined the linear fit slope to the elevation‐range dependence and required that the fitted slope error, $\delta m$, did not exceed 0.5 °/range gate and the slope was within a doubled error margin, $|m| \le 2 \cdot \delta m$.

The P2011 technique assumes that the elevation angles can be measured for any angle between $0^\circ$ and $90^\circ$. In reality, however, because the interferometer base exceeds the radar wavelength by a factor of three to five, the measured elevation angle has an uncertainty (e.g., Milan et al. 1997; McDonald et al. 2013; Ponomarenko et al. 2015, 2018) in that a single reported value of $\Delta$ can correspond to several propagation paths in the ionosphere. This is because the phase angle in the SuperDARN interferometric measurements is reported between $\pm\uppi$ while it actually can be different by multiples of $2\uppi$. SuperDARN radars report only the lowest possible elevations $\Delta_{{2\uppi}}$, from $0^\circ$ to $\sim 40^\circ - 45^\circ$ (Milan et al. 1997; McDonald et al. 2013; Ponomarenko et al. 2015, 2018). This ambiguity has not been addressed in the P2011 approach that we implemented in this study. Although it does not dramatically affect the measurements in a statistical sense because the events are infrequent, it might be critically important for some individual events.

Two aspects of the P2011 method are important to keep in mind. When actual elevation angles are larger than the SuperDARN limit angle $\Delta_{{2\uppi}}$, smaller elevation angles and, consequently, electron densities would be reported. This scenario might occur on the dayside, especially during high solar activity, when the ionospheric electron density can be high (e.g., Themens et al. 2014). There is also a limit on the minimum electron density that can be measured with the P2011 technique. This is because in a spherically stratified ionosphere even a zero-elevation ray enters plasma at a non-zero angle with respect to the contours of constant refractive index (electron density).

The above two effects imply that the P2011 technique has upper and lower limits on measurable $N_{\text{m}} F2$, on the order of $8 \times 10^{11} \;\text{m}^{ - 3}$ and $0.55 \times 10^{11} \;\text{m}^{ - 3}$, respectively (Ponomarenko et al. 2011). We note that for low RKN elevation angles ($\le 5^\circ$), statistical fluctuations inherent to SuperDARN signals can result in an erroneous $2\uppi$ shift in the interferometer phase angle difference, and the electron density inferred with the P2011 method would be significantly overestimated. This scenario is expected to occur on the nightside, especially in winter and generally at the far edges of echo bands.

## Geometry of observations and example of joint SuperDARN, CADI and RISR measurements

The P2011 technique was implemented for the RKN radar observations. One of the reasons for this radar selection was that it has been showing stable instrumental delay of the phase between the main and interferometer arrays. In addition, over a decade of its operation (since 2007), the radar has been showing one of the highest echo occurrence rates within the network (Ghezelbash, 2013; Koustov et al. 2019). The fact that independent $N_{\text{m}} F2$ measurements could be derived from the CADI ionosonde within the radar field of view was also an important consideration, although high-resolution ionogram scaling was not available at the time of the P2011 study. One of the instigating factors for the renewal of interest into testing of the P2011 method is the installation and successful operation of the RISR at Resolute Bay (Gillies et al. 2016) that, starting from 2016, has been providing high quality and detailed measurements of the electron density in the ionosphere for ~ 60 days in a year.

**Fig. 2:**
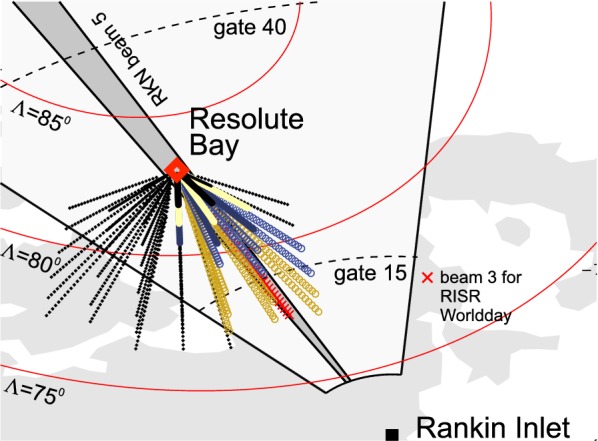
The field of view (FoV) of the Rankin Inlet (RKN) SuperDARN radar, large sector area, and RKN beam 5 (beam-like structure) oriented toward Resolute Bay (RB). At RB the CADI ionosonde and incoherent scatter radar RISR are stationed. Open circles are range gate locations for various RISR beams in the imaging mode (51 beams) of observations. Colored circles are range gate locations for the RISR beams in the imaging mode. Red crosses are range gate locations for the RISR beam 3 while the radar was operated in the world-day mode. Data from the shown colored beams were considered in this study

In the present work, routine observations of all three instruments were considered. Figure 2 shows the field of view (FoV) of the RKN radar and the orientation of its beam 5, which (in addition to adjacent beams 4 and 6) was used in this study. This beam crosses the Resolute Bay (RB) zenith. Figure 2 also shows the pierce points (at various heights) of multiple RISR radar beams whenever it was operating in its “imaging mode”, with a signal being transmitted and received in 51 beams. The radar has an electronically steerable array so that it can scan through pre-decided beam orientations on a pulse‐by‐pulse basis. In the imaging mode, the radar used a long pulse with a pulse length of 0.33 ms, corresponding to a range resolution of 50 km. The RISR beams were spread around the Resolute Bay zenith (Fig. 2) to provide detailed measurements in a relatively small spatial region. In this study, a subset of RISR data from 20 beams (large colored circles) was considered (for the beam numbers see Table 1). The selected beams (colored large circles in Fig. 2) are oriented toward the RKN radar so that the two instruments are observing approximately the same ionospheric region. In the imaging mode, the return signals were integrated over two intervals: 60 s and 300 s. We used the 300-s averaged electron densities. We also used RISR radar data collected in its “world-day mode” with 11 beams (Gillies et al. 2016). This mode of RISR operations is designed to measure ionospheric parameters over a much wider area which is critical for the reliable derivation of the plasma flow vectors from the line-of-sight velocity in multiple beams (Gillies et al. 2016). The radar range resolution was the same as in the imaging mode. For the world-day mode, only beam 3 data were considered. The reason is that the beam separation in this mode is much larger, while the orientation of beam 3 is almost exactly along the RKN beam 5. This allowed us to compare the RKN and RISR data in as close directions and spatial regions as possible. Even so, the spatial overlap between the regions of the RKN and RISR measurements is not ideal. It is not only that the RKN data were obtained in a band of ranges, gates 15–40 (see dashed lines in Fig. 2), but also because RISR electron density profiles were treated as being in the vertical direction while the actual data have been collected with tilted beams. The electron density was inferred from a multi-parameter fit to the measured autocorrelation functions and the measured total power. The RISR electron densities were routinely calibrated using the RB ionosonde (Themens et al. 2014).

**Table 1:** RISR and RKN radar beams selected for the comparison

	Beams RISR	Beams RKN
World-day	3	5
Imaging	4, 7, 8, 11, 14, 15, 18, 21, 22, 25, 28, 29, 32, 33, 36, 39, 40, 43, 46, 47	4, 5, 6

The electron density profiles from RISR measurements were often not very smooth. To infer the peak electron density, we analyzed each profile individually by considering all the electron density profile points close to the vicinity of the maximum, usually in the range of 200–350 km, and then made a fit to a Chapman-like function that had three free parameters, the scale height, the peak electron density, and its altitude. We note that the fitting provided values that were, typically, only slightly smaller (less than 10% different) than the absolute maximum electron density in the profiles.

The RKN radar was mostly operated with 1-min scans and used the standard 300 µs pulse length, providing 45-km resolution. An important feature of this radar is that its operating frequency has often been alternated between ~ 10 and ~ 12 MHz every new scan (especially in the last 5 years). The data from the standard FITACF output (Ponomarenko and Waters 2007; Ponomarenko et al. 2007) were analyzed. We note that there is some uncertainty in SuperDARN echo location on the order of 100 km (Yeoman et al. 2008). This uncertainty is not critical for the present work, as we consider RKN data in many gates (gates 15–40), covering > 500 km in range. The larger uncertainty is because no location for the echo band (involved in each 1-min electron density estimate) was determined. CADI observations are usually taken at a resolution of 1 to 5 min, but because of a desire to have long-term coverage, ionograms were manually scaled for every 30 min. For limited periods, 5-min ionograms were obtained.

## Example of three instrument observations

**Fig. 3:**
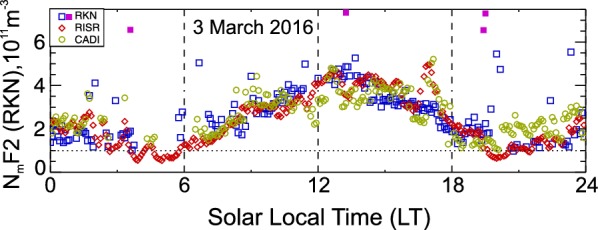
The F region peak electron density $N_{\text{m}} F2$ according to Rankin Inlet (RKN) HF radar elevation measurements in beams 4–6 (blue squares), CADI ionosonde (dark green open circles) data and RISR values (red diamonds) averaged over multiple beams (listed in Table 1). One full day of observations, 3 March 2016, is considered. For this plot, all RKN data with the antennae cross-phase deviation from its maximum possible value $\varPsi_{{\text{adj}}} > 250^\circ$ (see the text for the explanation) are shown by pink squares. This type of “anomalous” points was excluded from further analysis in this study. The dotted line denotes the $N_{\text{m}} F2$ of the instrumental low limit for RKN measurements

Figure 3 presents an example of joint 24-h-long measurements of $N_{\text{m}} F2$ with the three systems. RISR 5-min data are available continuously throughout the day. In these diagrams and other figures, we calculate Solar Local Time (LT) by assuming LT = UT − 6.5 h. CADI ionograms considered for this plot were scaled at a 5-min resolution. RKN data are shown by squares for every measurement available, but the data have two different colors: blue color for regular points and pink color for anomalous points. While analyzing the electron density estimates, we realized that some points were well above the generally expected electron density for the time of the day. These were presumably associated with measurements made when the antennae cross-phase deviation from its maximum possible value, $\varPsi_{{\text{adj}}}$ (Ponomarenko et al. 2018), was large. We remind the reader that $\varPsi_{{\text{adj}}}$ was introduced by Ponomarenko et al. (2018) as $\varPsi_{{\text{adj}}} = kd - \varPsi$, where *k* is the wave number for radio waves, *d* is the separation between the main and interferometer arrays ($kd$ is the maximum possible phase difference for observations along the radar boresight at zero elevation angle) and $\varPsi$ is the phase difference between the main and interferometer arrays. For this reason, such measurements have been discarded in this study (apart from Fig. 5 which serves to illustrate such data). The threshold for data removal was set at the level of $\varPsi_{{\text{adj}}} \ge 250^\circ$. Such points are shown as pink-colored squares in Fig. 3.

According to Fig. 3, $N_{\text{m}} F2$ varies significantly over the day, and the $N_{\text{m}} F2$ values from all three instruments vary consistently. The RKN radar shows an afternoon maximum. There are several data gaps at dawn (05–07 LT), at near noon and after 20 LT. At ~ 12 LT, the RISR electron densities are somewhat larger than those measured by CADI. In the dusk/midnight sector of 20–24 LT CADI shows larger electron densities than the other two systems.

In Fig. 3, a prolonged electron density minimum is seen between 04 and 07 LT where RISR was capable of continuously reporting the values, while the RKN radar only measured a few points. There were also very few CADI data points. The gap in HF radar and CADI ionosonde data indicates that these instruments cannot measure electron densities below $N_{\text{m}} F2\sim 1.0 \times 10^{11} \;\text{m}^{ - 3}$. This instrumental limitation is known from previous publications (e.g., Davies (1990) and Rawer (2013) for ionosondes and Davies (1990) and Ponomarenko et al. (2011) for HF radars).

The data presented in Fig. 3 indicate that RKN-based $N_{\text{m}} F2$ can be somewhat larger or smaller than that measured by CADI or RISR, but the agreement is generally reasonable. Figure 3 also indicates that the “anomalous” (pink) RKN-based points occur at the time of low electron density in the ionosphere as measured by RISR, one point is in the dawn sector and a couple of points are in the dusk sector. Reduced overall electron densities in the dawn sector and toward the midnight sector are generally expected (Themens et al. 2017, Fig. 2).

## Comparison of Rankin Inlet $N_{\text{m}} F2$ estimates and CADI ionosonde measurements

**Fig. 4:**
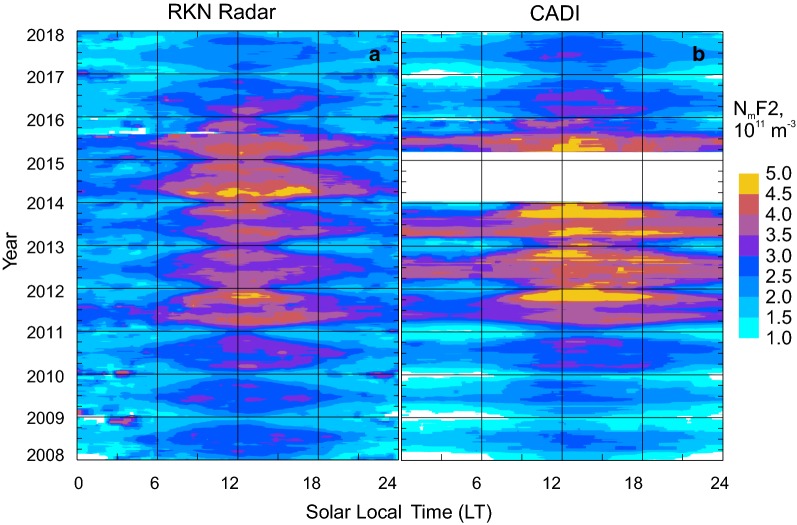
A contour plot of the F region peak electron density versus local time **a** for the Rankin Inlet (RKN) radar measurements in beams 2–6 and gates 15–40 and **b** according to CADI measurements at Resolute Bay. Solar local time was counted for the radar location, LT = UT − 6.5. Data at all RKN radar operating frequencies were considered

The Resolute Bay CADI has been in operation for almost 2 decades (Themens et al. 2017). The RKN radar data are available from 2008 onwards, allowing joint radar–ionosonde measurements that cover the period of 2008–2017. Figure 4 presents all of the data on $N_{\text{m}} F2$ collected by these systems as an occurrence plot on a local time–month plane, separately for each system. For this specific plot, the RKN measurements in beams 4–6, at gates 15–40, and at all radar operating frequencies were considered to ensure continuous coverage.

The plots of Fig. 4 are similar to those presented by Fig. 4 of Ponomarenko et al. (2011), but here we cover a significantly longer time period including the years of the solar cycle 24 maximum (2013–2015). The similarity between the plots of Fig. 4a, b is obvious. Indeed, electron density enhancements are centered around local noon and they are well seen in both plots. On both plots, electron density decreases in winter are obvious, especially in the midnight sector. For some years, for example in 2011, the electron density maxima are achieved at the equinoctial time, consistently in both data sets.

Differences between the RKN radar and CADI ionosonde $N_{\text{m}} F2$ values are also recognizable in Fig. 4a, b. One such difference is the larger nighttime RKN electron densities in most years (e.g., 2011). The other difference is generally larger daytime electron densities according to CADI (e.g., 2012–2015 data). However, in 2008–2010 (years of low solar activity), the near noon RKN electron densities are larger than those given by CADI. Interestingly, in 2017 (also a year of low solar activity), the relationship reverses.

**Fig. 5:**
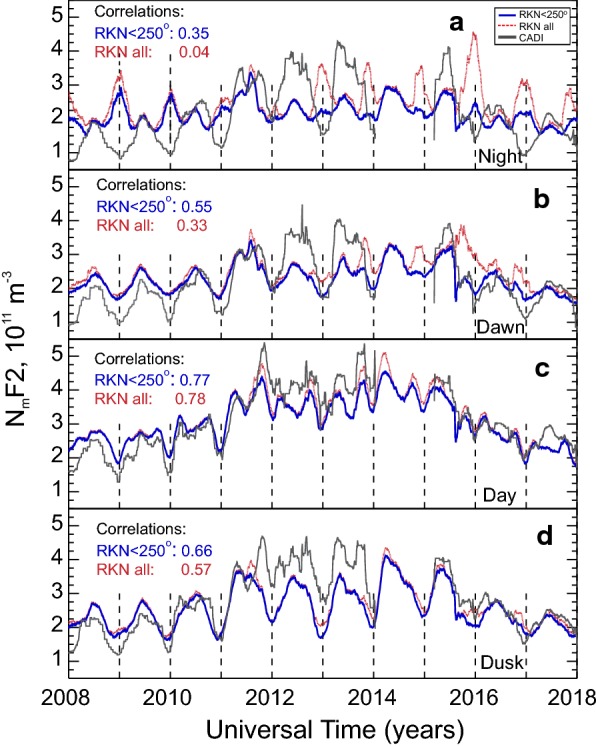
Line plots of the F region peak electron density $N_{\text{m}} F2$ averaged in four time sectors (**a** night: $0 \pm 3\,\text{LT}$; **b** dawn: $6 \pm 3\,\text{LT}$; **c** noon $12 \pm 3\,\text{LT}$; **d** dusk $18 \pm 3\,\text{LT}$) according to Rankin Inlet radar elevation measurements, red and blue lines. Red lines show $N_{\text{m}} F2$ estimates based on all radars records while blue lines represent $N_{\text{m}} F2$ estimates for limited data sets with discarded measurements for which the antennae cross-phase deviation from its maximum possible value $\varPsi_{{\text{adj}}}$ (Ponomarenko et al. 2018) was above value $250^\circ$. Radar data at all operating frequencies were considered. Grey lines are CADI-based data on $N_{\text{m}} F2$, averaged over the same local time intervals. Shown by numbers are the correlation coefficients between the radar and CADI curves. Lines for each instrument were obtained by applying $\pm$ 30-day boxcar sliding filter

To assess the data presented in Fig. 4a, b more explicitly, we present in Fig. 5a–d a series of line plots built for four time sectors: night ($0 \pm 3\;\text{LT}$), dawn ($6 \pm 3\;\text{LT}$), day ($12 \pm 3\;\text{LT}$) and dusk ($18 \pm 3\;\text{LT}$). Here the average $N_{\text{m}} F2$ is plotted for the entire period under consideration in each time sector. The RKN data are given for all measurements available (red lines) and for the data with cross-phase deviation from its maximum possible value $\varPsi_{{\text{adj}}}$ (Ponomarenko et al. 2018) being $< 250^\circ$. CADI data are shown by grey lines. Both CADI and RKN curves were obtained by applying $\pm 30$-day boxcar sliding filter (computing median values at each moment). To quantify the agreement between the data trends, the correlation coefficients between the curves are presented in the left-upper corner of each panel.

Let us first focus on the daytime observations, Fig. 5c, where agreement between the RKN and CADI data trends is best, as indicated by the correlation coefficients. Reasonable consistency, not only in trends but in values as well, is seen for some years (2011, 2015–2016). Both instruments show overall larger electron densities during years of high solar activity (2012–2014) which can be recognized by tracing the envelope of highest electron densities in each year. One can also see that anomalous points do not significantly affect the average electron densities, as the red curves are located only slightly above the blue curves, with the strongest effect at the equinoctial time in 2012 and 2013.

Trends for the dusk sector (Fig. 5d) are very similar but show a slightly worse correlation. The largest RKN $N_{\text{m}} F2$ underestimations are seen in 2012–2014.

For the dawn sector, shown in Fig. 5b, the red- and blue-colored curves are off the grey line and the trends divert, with the correlation coefficients between blue and grey curves and red and grey curves dropping down further to 0.55 and 0.33, respectively. In this case, one can hardly think of there being a linear relationship between the RKN and CADI measurements. The solar cycle effect is evident in the CADI data, but hardly recognizable in the RKN data.

Electron densities in the midnight sector (Fig. 5a) are expected to be low, especially during winter. While CADI (grey line) densities behave as expected, RKN observations show the opposite, with spikes of electron density in the winter. From 2013 onward, the elimination of high-phase RKN data greatly improves overall agreement with CADI. This shows that the high-phase RKN data have the most significant impact when electron densities are low. We also see the anti-correlation in 2008–2009, but here the elimination of high-phase data gives only a very small improvement. Also, significant RKN electron density underestimations are seen in the summers of 2011–2013.

**Fig. 6:**
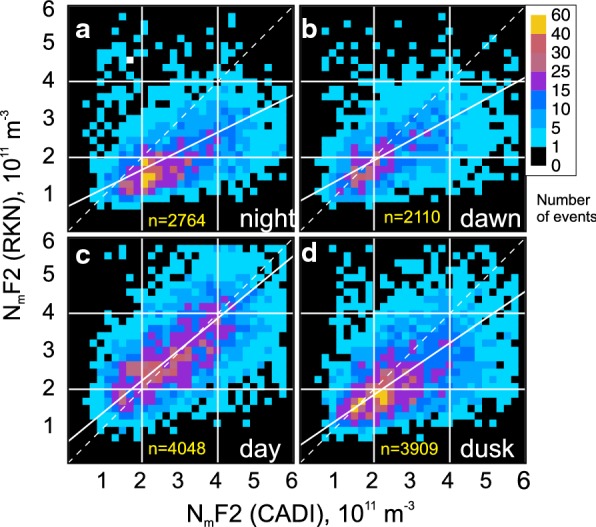
Scatter plots of the Rankin Inlet $N_{\text{m}} F2$ estimates versus CADI-based $N_{\text{m}} F2$ for matched moments and four local time sectors: **a** night $0 \pm 3\,\text{LT}$; **b** dawn: $6 \pm 3\,\text{LT}$; **c** noon $12 \pm 3\,\text{LT}$; **d** dusk $18 \pm 3\,\text{LT}$. Radar data in 2008–2017 at all operating frequencies were considered. The total number of available points *n* is shown at the bottom of each panel (yellow). In all panels, the solid lines are the lines of best linear fit

One criticism of the above data trend analysis is that the amount of radar and ionosonde data points involved differ significantly and the measurements are not simultaneous. To further the comparison, we present in Fig. 6a–d scatter plots of the RKN-based $N_{\text{m}} F2$ estimate versus CADI-based $N_{\text{m}} F2$ for matched moments. Each plot in Fig. 6a–d is for a corresponding local time sector of Fig. 5a–d. Here the RKN data at all operating frequencies were considered.

The scatter plots in Fig. 6a–d, for nearly coincident measurements (a 1-min RKN measurement being < 1 min apart from a CADI measurement), show that the RKN-based $N_{\text{m}} F2$ values are smaller than those measured by CADI, with the exception of daytime. The effect is stronger for the nighttime. For daytime, the RKN $N_{\text{m}} F2$ values tend to be close to those measured by CADI. Overall, however, one can conclude that there is a reasonable agreement between the two instruments, particularly for the daytime. This judgment is based on good data clustering not far away from the bisector of perfect agreement. In addition, the linear fits to the data clouds in Fig. 6a–d are 0.49, 0.55, 0.82 and 0.68, respectively. The correlation coefficients are not high ranging from 0.4 to 0.6.

## Comparison of Rankin Inlet $N_{\text{m}} F2$ estimates and RISR ISR values

**Fig. 7:**
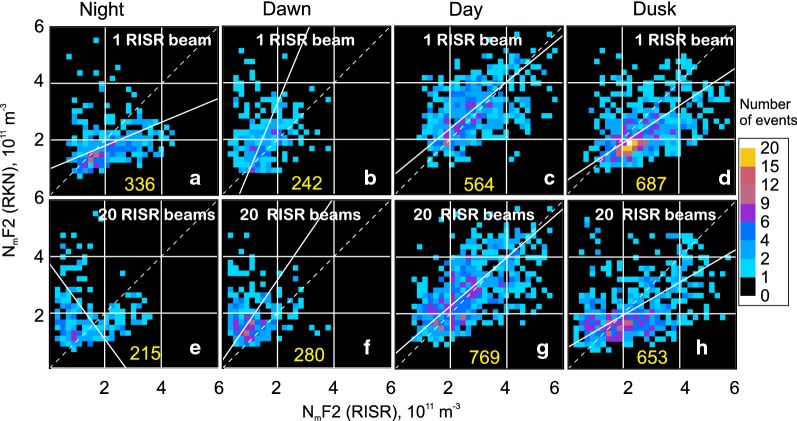
Scatter plots of the F region peak electron density $N_{\text{m}} F2$ inferred from Rankin Inlet (RKN) radar measurements (beams 4–6) versus $N_{\text{m}} F2$ inferred from RISR measurements. For RKN, data at all operating frequencies were considered. **a–h** are for RISR observations in the world-day and imaging modes, respectively, with averaging over the beams as described in the text. The local time sectors are the same as for those for Fig. 6. The total number of available points is shown at the bottom of each panel (yellow). In all panels, the solid lines are the lines of the best linear fit

RKN and RISR plots of joint electron density measurements, similar to that of Fig. 6a–d, were produced for about 60 events in 2016 for which RISR was operational for at least 2 h. The plots were built from the world-day and imaging modes of RISR operation separately, Fig. 7. Since RISR data were given only for ~ 60 days and with a 5-min temporal resolution, the number of joint measurements is not as large as for the RKN-CADI comparison of Fig. 6, especially after the data were split onto four time sectors; however, the statistics are sufficiently large to infer trends.

Scatter plots of Fig. 7 show a general agreement between the instruments although the degree of agreement varies. One obvious feature in Fig. 7 is smaller RKN electron densities at night and dusk (Fig. 7a, d, e, h) as evidenced by the cloud of points located below the bisector of perfect agreement. The points for the daytime and dawn sectors (Fig. 7b, c, f, g) are spread significantly so that the data clouds are of a circular shape. They are, however, centered on the bisector of perfect agreement with the daytime data being slightly shifted to higher RKN values. The slopes of the best fit line in the case of Fig. 7a–d are 0.42, 2.4, 0.82 and 0.67, respectively.

For the case of Fig. 7e–h, the slopes are − 1.36, 1.46, 0.86 and 0.56, respectively. The slopes for daytime and dusk (the last two numbers for each data set) are consistent with the slopes for the RKN-CADI comparisons of Fig. 6. They support our judgment on the reasonable agreement between the instruments. Slopes for the nighttime and dawn data differ greatly from expectations. The reasons for this result are discussed below.

The prominent feature that stands out in Fig. 7 is the occurrence of points located well above the bisector of perfect agreement whenever the RISR electron densities are low, below ~ $N_{\text{m}} F2\sim 1 \times 10^{11} \;\text{m}^{ - 3}$ (Fig. 7a, e and especially Fig. 7b, f). These are in abundance for the nighttime and dawn plots. This effect can also be identified in Fig. 5a where nighttime RKN and CADI data are compared. We remind the reader that the RKN data of Fig. 7 include only measurements with the cross-phase deviation from its maximum possible value $\varPsi_{{\text{adj}}}$ (Ponomarenko et al. 2018) being below $250^\circ$. One can conclude that this limitation is insufficient to remove all erroneous RKN measurements with the currently implemented electron density estimation algorithm.

Although it is not readily apparent, such anomalous RKN points are present in the RKN-CADI data set of Fig. 6, but their total number is small in the large body of measurements. Another important issue here is that both ionosondes and HF radars are unable to detect echo signals at low electron densities, contrary to ISRs.

Data of Fig. 7 indicate that the anomalous points are more frequent for the imaging mode of RISR operation. We believe that this is purely owing to the relatively small number of data points. Aside from this feature, the scatter plots of Fig. 7 for the two modes of RISR measurements (top and bottom rows) look similar. Generally, similar plots for the single (Fig. 7a) and multiple beam (Fig. 7b) RISR measurements indicate that SuperDARN data, based on the analysis of echo signals from spatial regions of ~ 200 km size, is a reasonable representation of the electron density in a broad area around the Resolute Bay zenith most of the time.

## Assessment of RKN electron density estimates with quantile analysis

**Fig. 8:**
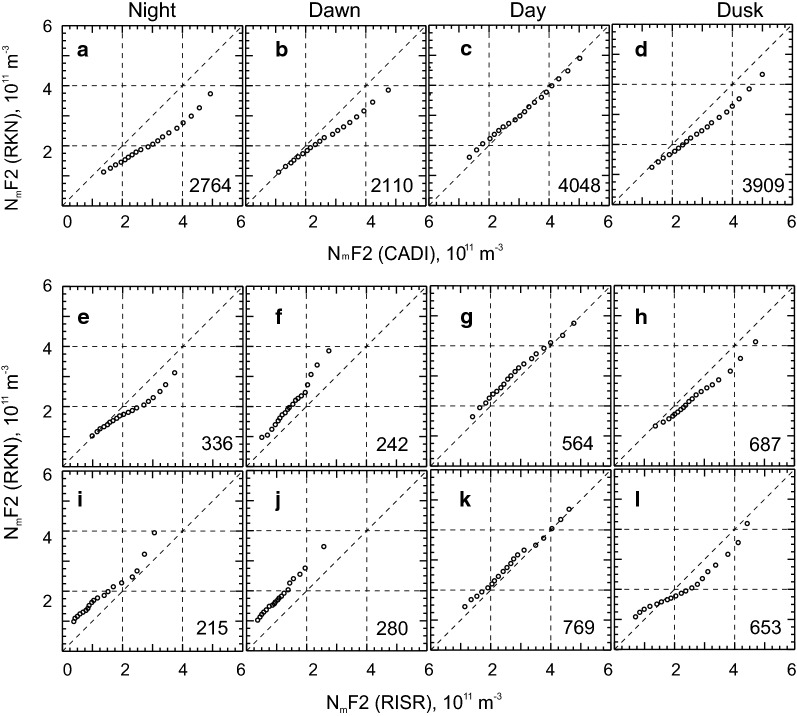
Quantile–quantile (Q–Q) plots of the $N_{\text{m}} F2$ nearly simultaneous measurements by the RKN radar and the CADI ionosonde or the RISR radar for various time sectors and experiments. **a**–**d** are for the RKN-CADI joint data set, **e**–**h** are for the RKN-RISR 1 beam joint data set and **i**–**l** are for the RKN-RISR 20 beams joint data set. The total number of points for each data set is shown at the bottom of the plots

To alternatively assess the quality of the $N_{\text{m}} F2$ estimates from RKN measurements, we performed the additional analysis by producing the so-called quantile–quantile (Q–Q) plots of the RKN and CADI/RISR data. We adopted an approach similar to that of Tindale and Chapman (2017). The data for each of the instruments were first ranked according to reported values of the electron density and then the quantiles were selected between 5 and 95% of the total number of points with a step of 5%. For each quantile level, the electron density value inferred from the RKN measurements was paired with a similarly computed value from another instrument, CADI or RISR. All the point pairs were then placed on a 2-D plane with the RKN values being plotted along the *Y* axis, Fig. 8, forming the Q–Q plots. We selected the same data sets as those considered in Figs. 6 and 7 and placed them in a similar fashion to aid in comparison.

The Q–Q plots of Fig. 8c, g, k show that the daytime electron densities, corresponding to various quantiles, are collocated with the bisector of the perfect agreement for both the RKN-CADI and the RKN-RISR data sets (in both modes of the RISR operation). This indicates that the data distributions for independent instruments are almost the same. Data of Fig. 8 in other time sectors show departures of the points from the bisector of perfect agreement.

For the dusk observations, Fig. 8d, h, l, the points are somewhat off the bisector but align reasonably with it for $N_{\text{m}} F2 = (1 - 3) \times 10^{11} \;\text{m}^{ - 3}$. For larger CADI or RISR densities, the RKN values are systematically smaller. This indicates that the data distributions deviate in the tails of the distributions, with the RKN values being systematically smaller. This effect had been mentioned earlier and can be recognized in the scatter plots of Figs. 6, 7.

The RKN-CADI dawn plot of Fig. 8b is similar to that of the dusk plot of Fig. 8c except for the deviations from the bisector at large electron densities are slightly stronger. For nighttime, Fig. 8a, the deviations of the RKN and CADI distributions are evident at just about any electron density.

The RKN-RISR data for the dawn and nighttime, Fig. 8f, i, g, show another type of difference. The points here are located well above the bisector of perfect agreement, indicating the presence of higher-value records in the RKN data sets. This is consistent with the scatter plots of Fig. 7.

We also performed the Q–Q analysis by considering all the RKN data on the electron density in 2008–2017 and all the CADI electron density data that are available to us. In this analysis, matching in time was not required. We found that the Q–Q plots for various time sectors looked very much similar to those shown in Fig. 8a–d indicating the robustness of the results for the joint RKN-CADI data set. The Q–Q plots for the entire RKN data set and the entire RISR data set showed differences similar to those shown in Fig. 8i–l.

## Does the radar frequency of the SuperDARN measurements affect the electron density estimates?

As we have already mentioned, the RKN radar has often been operating in a dual frequency mode, switching the frequency of transmission between 10 and 12 MHz every other scan (every other minute). Although the $N_{\text{m}} F2$ estimates with the RKN radar should be, ideally, independent of the operating frequency, some differences might be expected. One reason is that the bands of echoes involved in electron density estimates do not typically coincide. The other potential factor is the difference in the scattering height. For this reason, here we investigate the effect of radar operating frequency on the $N_{\text{m}} F2$ estimates.

**Fig. 9:**
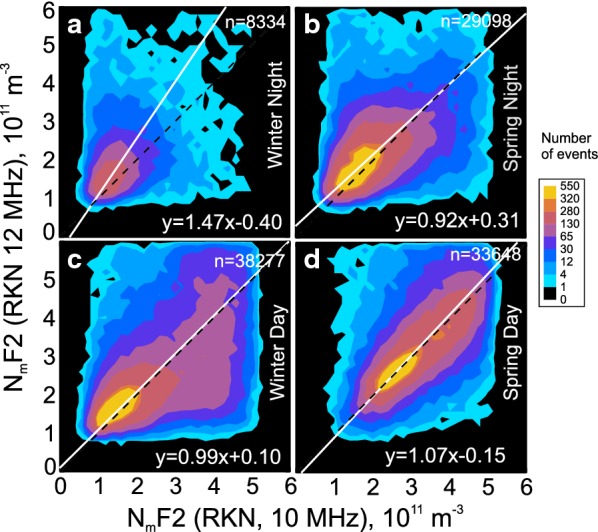
Contour plots of the F region peak electron density $N_{\text{m}} F2\text{(}12\text{)}$ inferred from Rankin Inlet (RKN) radar measurements (beams 4–6) at 12 MHz $N_{\text{m}} F2\text{(}10\text{)}$ inferred from RKN measurements at 10 MHz. **a**, **b** Are for nighttime observations in winter and spring, respectively. **c**, **d** Are for daytime observations in winter and spring, respectively. All records in 2008–2017 have been considered. The nighttime and daytime sectors were selected as $0 \pm 3\,\;\text{LT}$ and $12 \pm 3\;\text{LT}$, respectively. The total number of available points *n* is shown at the upper-right corner of each panel. In all panels, the solid lines are the lines of the best linear fit

Figure 9 compares $N_{\text{m}} F2$ inferred at 12 MHz versus $N_{\text{m}} F2$ inferred at 10 MHz for measurements with a time separation of 1 min. Only daytime and nighttime plots are presented because they reflect the extremes that we identified in the plots for all time sectors. For the daytime, the consistency between the data is evident. Similar results are seen in plots for dusk and dawn (not presented here). Data for nighttime (Fig. 9a, b) show noticeable differences. Here the electron densities inferred from 12 MHz measurements are slightly larger than those inferred from 10 MHz measurements. Similar, but weaker, inconsistencies are seen for nighttime winter observations in the dawn and dusk sectors (data are not presented here). We note that these are the periods with generally lowest electron densities and strongest north–south electron density gradients in terms of season and local time.

## On the improvements of electron density estimates from solely RKN data

The data of Figs. 6 and 7 show that RKN-based electron densities differ from those measured by the CADI ionosonde and the RISR radar, both of which are well established instruments used to characterize the electron density distribution in the ionosphere. To use only RKN radar for $N_{\text{m}} F2$ estimation, the HF measurements have to be adjusted to align with the established observations of the F layer maximum. Here we propose a simplified recipe for such an adjustment.

**Fig. 10:**
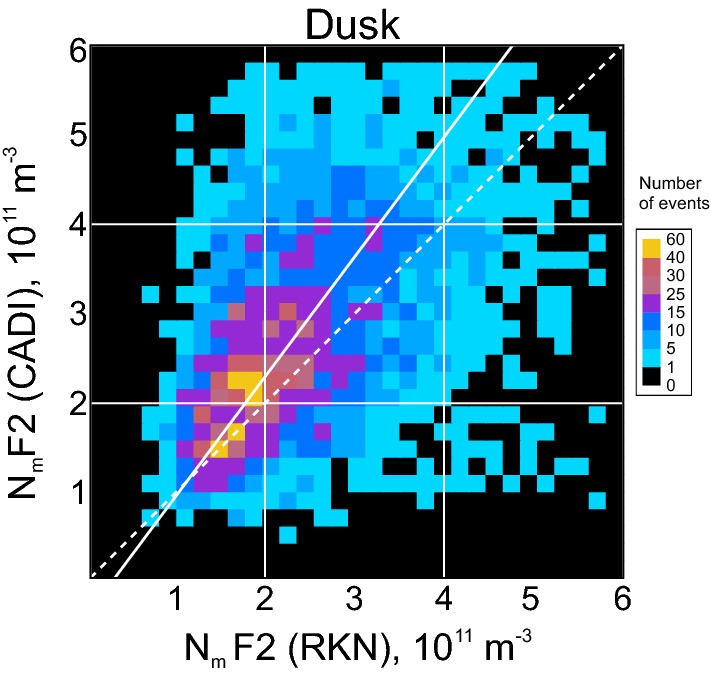
Scatter plot of the F region peak electron density $N_{\text{m}} F2$ inferred from Rankin Inlet (RKN) radar measurements (beams 4–6) versus $N_{\text{m}} F2$ inferred from CADI measurements. All dusk sector observations in 2008–2017 were considered. The white line is the linear fit line to the data, as described in the text

Figure 10 illustrates the approach. We show here the scatter plot of the RKN $N_{\text{m}} F2$ versus CADI $N_{\text{m}} F2$, for the dusk observations. This is a redrawn Fig. 6d. Similar plots have been considered for all other panels of Figs. 6 and 7.

To characterize the plot in Fig. 10, we conducted a linear fit by minimizing the standard deviations of all the point distances from the linear fit line in the direction perpendicular to the fit line. This method of fitting was done because both instrument measurement techniques have some uncertainties. Our goal was to establish the relationship of the type $N_{\text{m}} F2\text{(CADI)} = a \cdot N_{\text{m}} F2\text{(RKN)} + b$. Such a relationship allows one to “calibrate” the RKN measurements, for the dusk sector. The coefficients of the fit lines, for Fig. 10 and similar plots for other time sectors (not presented here), are given in Table 2 along with the correlation coefficients of the scatter plots and the total number of points involved. The slopes of the lines are all above 1.0 signifying a general tendency for the RKN measurements to underestimate the peak electron density in the ionosphere, as measured by CADI. The negative intercepts indicate that RKN does not accurately measure very low densities, as we have previously discussed. Application of these empirical relationships to individual measurements should provide a better quality estimate of $N_{\text{m}} F2$ in the RB zenith.

**Table 2:** Coefficients of a linear fit $N_{\text{m}} F2\text{(CADI)} = a \cdot N_{\text{m}} F2\text{(RKN)} + b$ to the scatter plots of RKN electron density versus CADI (Fig. 6)

	*a*	$b\,\text{(} \times 10^{11} \;\text{m}^{ - 3} \text{)}$	Correlation coefficient	Number of points
Night	1.92	− 0.93	0.39	2764
Dawn	1.56	− 0.83	0.45	2110
Day	1.11	− 0.35	0.59	4048
Dusk	1.42	− 0.53	0.47	3909

Similar fitting has been done for the joint RKN-RISR data. We were seeking the dependence of a type $N_{\text{m}} F2\text{(RISR)} = a \cdot N_{\text{m}} F2\text{(RKN)} + b$. The fit line slopes were found to be strongly affected by the erroneous RKN estimates at low ionospheric electron densities (as measured by RISR) below $\sim 1 \times 10^{11} \;\text{m}^{ - 3}$. On our second attempt, we excluded all the points with such low RISR electron densities. The coefficients of these second-attempt fit lines are given in Table 3 along with the correlation coefficients of the scatter plots and the total number of points involved in each plot. The slopes of the lines are close to or above 1.0 for daytime and dusk observations, indicating a general tendency for the RKN electron densities to underestimate the peak electron density in the ionosphere, equatorward of the RB location. We note that the correlation coefficients are very low (< 0.3) for the nighttime and dawn observations, such that the fit lines here do not have practical significance and implying that improvement of the SuperDARN-based electron density estimates is needed for the cases of low F region peak electron densities.

**Table 3:** Coefficients of a linear fit $N_{\text{m}} F2\text{(RISR)} = a \cdot N_{\text{m}} F2\text{(RKN)} + b$ to scatter plot of RISR electron density versus RKN electron density for 1 beam (first 4 lines) and 20 beam (last 4 lines) RISR measurements (Fig. 7)

	*a*	$b\text{(} \times 10^{11} \;\text{m}^{ - 3} \text{)}$	Correlation coefficient	Number of points
Night, 1 beam	2.38	− 1.84	0.23	304
Dawn, 1 beam	0.53	0.62	0.28	175
Day, 1 beam	0.96	− 0.13	0.49	563
Dusk, 1 beam	1.56	− 0.64	0.44	680
Night, 20 beams	− 0.44	2.84	− 0.14	111
Dawn, 20 beams	0.60	0.44	0.15	142
Day, 20 beams	1.09	− 0.41	0.56	743
Dusk, 20 beams	1.40	− 0.32	0.47	546

## Discussion and conclusions

An important general conclusion from the comparisons of the RKN-based electron densities $N_{\text{m}} F2$ and those measured by CADI and RISR is that the RKN estimates are reasonable most of the time, as indicated by good clustering of the data along the expected bisector line on the plots of Figs. 6 and 7. The data showed better agreement for daytime conditions and significant differences at nighttime.

For daytime conditions, an interesting very minor effect in the reported data is the RKN electron density overestimation during daytime (Figs. 6, 7). This can be explained by the fact that under enhanced electron density, the RKN echo bands shift to lower latitudes so that the RKN electron density estimates are related to somewhat lower latitudes than the Resolute Bay location for CADI and, to some extent, than the RISR latitudes of measurements. This increase of the electron density with decreasing latitude at lower polar cap latitudes is expected at daytime (Themens et al. 2017). It can be as large as $\text{(}0.2 - 0.5\text{)} \times 10^{11} \;\text{m}^{ - 3}$ over two degrees of latitude (potentially possible separation between the RKN and CADI echo detection zones).

We also found a slight dependence of RKN density measurements on radar frequency; electron densities at 12 MHz are slightly larger, on average, than those at 10 MHz (Fig. 9) for winter nighttime conditions but not for the daytime conditions, Fig. 9. We expect that 12 MHz echoes would come at somewhat larger ranges as compared to those at 10 MHz, statistically speaking. The typical latitudinal difference would be ~ 100 km. However, one can find cases with spatial separation between 10 and 12 MHz echo detection zones up to 5 range gates, i.e., 2° of latitude. An important feature of the ionosphere equatorward of Resolute Bay is that the maximum electron density can actually decrease toward lower latitudes by $\text{(}0.1 - 0.2\text{)} \times 10^{11} \;\text{m}^{ - 3}$ over two degrees of latitude at nighttime (Themens et al. 2017). Although the effect seems to be weak, it can explain partially the reason for the difference in the peak electron density inferred from 10 and 12 MHz measurements.

Our comparison for the nightside and morning hours showed occasional strong disagreements with CADI and, especially, RISR. One interesting effect is the generally smaller electron densities in RKN measurements during nighttime (Figs. 6, 7). Several effects very likely contribute to this. The first one is the fact that the RKN electron density derivation procedure considers elevation angles for the Pedersen rays propagating at the top of the F layer, i.e., slightly above the height of the electron density peak. Thus, the real maximum electron densities at the F layer peak are slightly larger than the reported ones. The second potentially contributing factor is an assumption of the fixed height of the scatter at 250 km (Eq. 1). Since the nighttime heights of the F layer can be as high as 400 km, the real values of the peak electron density can be larger than those inferred by assuming a 250-km height, by a factor of up to ~ 1.15, which one can estimate from equations of Ponomarenko et al. (2011). The impact of the fixed height assumption for the daytime electron density estimates is not that important since the real heights of the daytime scatter are much closer to 250 km.

We hypothesize that the lower RKN electron densities are also due to the averaging effect in HF radar measurements. While CADI and RISR are detecting signals from localized regions with the strongest electron density, the elevation angles in RKN measurements are “averaged” over at least five radar gates (225 km) so that any localized electron density enhancement is smoothed out. The smoothing effect is expected to be stronger for ionospheric conditions with higher patchiness and poorer propagation conditions. Out of all time sectors, this is very likely to occur at winter nighttime because here the ionosphere is depleted and highly patched. It is not a surprise then that the RKN under-estimation effect is stronger at winter nighttime.

The data presented show occasional dramatic RKN electron density overestimation at low real ionospheric electron densities (according to RISR); these measurements were classified as “anomalous points” (Figs. 3, 5, 7). We indicated that this happens due to the incorrect $2\uppi$ wrapping of the phase angle in interferometric measurements. The number of these points is not usually significant, as we learned from the RKN data set analysis. For example, we estimated that these points make up ~ 6% of our data for February 2016. In terms of the time sector, these points were mostly seen at the nighttime and dawn and in the afternoon in 2016 (example in Fig. 3). For other years of observations, the number of such points and their preferential occurrence time varied. Figure 5 shows that these points have the most impact on nighttime data, and especially when density is low. The issue requires further investigation. Figure 7 indicates clearly that despite our efforts, quite a few erroneous measurements are still in the database implying that simply removing all measurements with the antennae cross-phase deviation from its maximum possible value $\varPsi_{{\text{adj}}} > 250^\circ$ does not fully resolve the problem. We are planning to address the issue in the future.

We can summarize the results of this study as follows:

1. The RKN radar estimates of the electron density at the F region peak in the RB area are reasonably consistent, most of the time but not always, with measurements by the CADI ionosonde measuring the electron density in the zenith of RB and the RISR ISR measuring electron density in much broader area mostly southward of RB.
2. The RKN peak electron density estimates are in better agreement with CADI and RISR for daytime and for electron densities in the range of $N_{\text{m}} F2\sim \text{(}1 - 3\text{)} \times 10^{11} \;\text{m}^{ - 3}$ at dusk. For daytime, there is a very minor overestimation effect. For other time sectors, there is a tendency for the RKN electron density to be underestimated.
3. There is a very subtle tendency for the $N_{\text{m}} F2$ inferred from 12 MHz measurements to be larger than that inferred from 10 MHz measurements during nighttime, away from summer.
4. The RKN peak electron density estimates for nighttime and dawn conditions when the electron densities are below $N_{\text{m}} F2\sim 1 \times 10^{11} \;\text{m}^{ - 3}$ can often be erroneous due to $2\pi$ shifts of the phase in interferometric measurements. In the present study, to diminish the role of this effect, some potentially affected RKN data were discarded.
5. The results imply that SuperDARN dayside and duskside electron density estimates are justifiable for their use in statistical studies, such as those focused on seasonal and solar cycle effects. Straight application of the SuperDARN electron densities for the analysis of individual events is less certain as local anomalies do occur, and they have to be investigated on a case-by-case basis.

## Data Availability

SuperDARN data can be obtained from https://superdarn.ca. RISR data are available at Madrigal database http://madrigal.phys.ucalgary.ca or http://data.phys.ucalgary.ca. CADI data are available for download at http://chain.physics.unb.ca.

## Abbreviations

CADI: Canadian Advanced Digital Ionosonde
e-CHAIM: Empirical Canadian High Arctic Ionospheric Model
FoV: Field of view
GS: Ground scatter
HF: High frequency
IMF: Interplanetary Magnetic Field
IRI: International Reference Ionosphere
IS: Ionospheric scatter
ISR: Incoherent scatter radar
LOS: Line-of-sight
LT: Local time
MHz: Megahertz
RB: Resolute Bay
RKN: Rankin Inlet
SuperDARN: Super Dual Auroral Radar Network
RISR: Resolute Incoherent Scatter Radar-Canada
